# Use of transcranial magnetic stimulation (TMS) for studying cognitive control in depressed patients: A systematic review

**DOI:** 10.3758/s13415-024-01193-w

**Published:** 2024-05-21

**Authors:** Ana Hernández-Sauret, Ona Martin de la Torre, Diego Redolar-Ripoll

**Affiliations:** 1 Cognitive Neurolab, Faculty of Health Sciences, Universitat Oberta de Catalunya (UOC), Rambla del Poblenou 156, Barcelona, Spain; 2 Instituto Brain360, Unidad Neuromodulación y Neuroimagen, Calle Maó 9, Barcelona, Spain

**Keywords:** Transcranial magnetic stimulation, Noninvasive brain stimulation techniques, Cognitive control, Major depressive disorder

## Abstract

Major depressive disorder (MDD) is a debilitating mental disorder and the leading cause of disease burden. Major depressive disorder is associated with emotional impairment and cognitive deficit. Cognitive control, which is the ability to use perceptions, knowledge, and information about goals and motivations to shape the selection of goal-directed actions or thoughts, is a primary function of the prefrontal cortex (PFC). Psychotropic medications are one of the main treatments for MDD, but they are not effective for all patients. An alternative treatment is transcranial magnetic stimulation (TMS). Previous studies have provided mixed results on the cognitive-enhancing effects of TMS treatment in patients with MDD. Some studies have found significant improvement, while others have not. There is a lack of understanding of the specific effects of different TMS protocols and stimulation parameters on cognitive control in MDD. Thus, this review aims to synthesize the effectiveness of the TMS methods and a qualitative assessment of their potential benefits in improving cognitive functioning in patients with MDD. We reviewed 21 studies in which participants underwent a treatment of any transcranial magnetic stimulation protocol, such as repetitive TMS or theta-burst stimulation. One of the primary outcome measures was any change in the cognitive control process. Overall, the findings indicate that transcranial magnetic stimulation (TMS) may enhance cognitive function in patients with MDD. Most of the reviewed studies supported the notion of cognitive improvement following TMS treatment. Notably, improvements were predominantly observed in inhibition, attention, set shifting/flexibility, and memory domains. However, fewer significant improvements were detected in evaluations of visuospatial function and recognition, executive function, phonemic fluency, and speed of information processing. This review found evidence supporting the use of TMS as a treatment for cognitive deficits in patients with MDD. The results are promising, but further research is needed to clarify the specific TMS protocol and stimulation locations that are most effective.

## Introduction

Major depressive disorder (MDD) is one of the most prevalent mental disorders worldwide (5% of adults) and one of the most disabling (Gutiérrez-Rojas et al., 2020). It affects people of all ages, sex, and ethnicities. It has been listed as the highest cause of disease burden within mental disorders (Anon, 2022). In the past few years, because of the COVID-19 pandemic among other situations, there has been an increase in patients suffering from MDD (Santomauro et al., 2021). In the first year of the COVID-19 pandemic, the global prevalence of anxiety and depression increased by 25%, according to a scientific brief released by the World Health Organization (WHO, 2022).

This disorder is usually characterized by persistent sadness, loss of interest or pleasure, decrease or increase in appetite nearly every day, a slowing down of thoughts and a reduction of physical movement, loss of energy, feeling of worthlessness and inappropriate guilt, and recurrent thought of death (American Psychiatric Association and American Psychiatric Association, 2013). However, this disorder impairs more than just the emotional domain.

Major depressive disorder is associated with broad cognitive deficits, evidenced by difficulties in working memory, executive function, and processing speed among other functions (Chakrabarty et al., 2016; LeMoult & Gotlib, 2019). Given the widespread prevalence of this disorder in our society, there exists an extensive body of literature and research dedicated to it. However, most of the studies have focused their attention on the clinical manifestations, neglecting the cognitive deficit and its importance in the development of MDD. There is evidence that cognitive impairment persists even after symptom remission, contributing to ongoing occupational and social dysfunction and thereby the risk of recurrent depressive episodes (Baune & Air, 2016). Hence, emphasizing the need to go further in the study of cognition in depressed patients.

Cognitive control is a capacity that allows us to use our perceptions, knowledge, and information about our goals and motivations to shape the selection of a goal-directed action or thought (Ripoll, 2021). To carry out goal-oriented behaviour, we need to keep task-related information active and nontask-related information inhibited. Functions, such as working memory and attention, are needed to keep task-relevant information active and to manipulate that information to achieve our goals. Cognitive control is the primary function of the prefrontal cortex (PFC). This area is an assembly of cortical regions interconnected with almost all cortical and subcortical structures involved in emotion and cognition. The lateral regions of the PFC, particularly the left dorsolateral PFC, are thought to be responsible for these PFC-related cognitive functions (Teffer & Semendeferi, 2012). Neuroimaging studies in depressed subjects have reported an abnormal function of the prefrontal areas (Shen et al., 2015; Ye et al., 2015). This alteration of the DLPFC network is associated with cognitive deficits in patients diagnosed with MDD (Zhang et al., 2018).

In terms of treating MDD, the primary treatments are psychological therapy and pharmacological treatment. One of the most common forms of psychological therapy is cognitive behavioral therapy (CBT), which is a goal-oriented approach that assists individuals in recognizing and altering negative thought patterns and behaviors that contribute to emotional distress (Cuijpers et al., 2023). The second consists of drugs, such as selective serotonin reuptake inhibitors (SSRI) or tricyclic antidepressants (TCA). However, this is not suitable or well tolerated by everyone. Between 15–30% of patients receiving pharmacological treatment do not respond adequately (Petersen et al., 2001). Hence, these patients are generally referred to as treatment-resistant depression (TRD) (Gaynes et al., 2020) and represent over half of the total annual costs associated with the treatment of this disorder (Petersen et al., 2001) . As a result of this lack of effectiveness for the treatment of MDD, alternative techniques have emerged, such as transcranial magnetic stimulation (TMS) or transcranial direct current stimulation (tDCS).

We focused our review on TMS for several reasons. From a physiological standpoint, it is essential to differentiate the mechanisms of action between TMS and tDCS. Transcranial magnetic stimulation involves the utilization of electromagnetic coils to generate magnetic fields capable of stimulating specific brain regions. This stimulation modulates cortical excitability either increasing or attenuating it. Conversely, tDCS operates by administering low-intensity electrical currents directly to the scalp, thereby exerting influence on neuronal activity. The modus operandi of tDCS revolves around altering the neuronal threshold, rendering firing more or less likely. Notably, by manipulating the excitability of the dorsolateral prefrontal cortex (DLPFC)—the target area in MDD treatment—it is possible to adjust functional connectivity more reliably compared with simply changing neuronal thresholds (Lefaucheur, 2019).

In addition, conventional tDCS employs an anode and a cathode, causing electrons to traverse the brain from the anode to the cathode. This process makes the stimulation less precise, as the electric current disperses more diffusely through the brain tissue. Moreover, studies have shown that intensity levels of tDCS did not significantly affect the outcome (Agboada et al., 2019). However, TMS is more focal and can precisely target specific brain areas for stimulation. By adjusting the parameters of the magnetic field, such as intensity, duration, and frequency, TMS can modulate neuronal activity in targeted brain regions with high precision (Sparing & Mottaghy, 2008; Klomjai et al., 2015; Nitsche et al., 2015).

Finally, the United States Food and Drug Administration (FDA) has approved TMS to treat MDD. Thorough clinical trials that validated the product’s safety and effectiveness led to its clearance. tDCS has shown potential in some studies, but when compared to TMS, it might not have as much clinical proof or regulatory support for treating MDD (Center for Devices and Radiological Health, 2018).

Transcranial magnetic stimulation is based on the scientific principle of electromagnetic induction. The equipment uses a high current pulse generator able to produce a discharge current of several thousand amperes that flows through a stimulating coil, generating a brief magnetic pulse with field strengths up to several Teslas. This magnetic pulse, by the principles of electromagnetism, produces secondary electric fields in the opposite direction to the field generated by the coil (Faraday’s Law). If the coil is placed on the head of a subject, the magnetic field created undergoes little attenuation by extracerebral tissues (scalp, cranial bone, meninges, and cerebrospinal fluid layer) and is able to induce an electrical field sufficient to depolarize (or hyperpolarize) superficial axons and to activate neural networks in the cortex (Chervyakov et al., 2015). Transcranial magnetic stimulation exerts its influence on the brain through an interplay of physics and biology. At the cellular level, TMS interacts with neurons in two primary ways, depending on the stimulation intensity. When the magnetic field is strong (high-intensity stimulation), it can directly trigger action potentials, the electrical signals that neurons use to communicate. This process mimics the natural firing of neurons within the brain. When examining low-intensity stimulation, the situation gains complexity. Rather than directly inducing neuronal firing, low-intensity TMS functions as a modulator, subtly affecting their activity. Transcranial magnetic stimulation can alter a neuron’s resting membrane potential (its electrical state) or its connections to other neurons, making them more or less excitable (Gangitano et al., 2002). While the exact cellular mechanisms remain under investigation, the leading theory suggests that the electric fields generated by the TMS coil interact with the charged membranes of neurons, particularly at bends or junctions in their fibers. This interaction likely influences the flow of ions within the neurons, ultimately affecting their firing patterns and information processing capabilities. The impact of TMS extends beyond individual neurons, influencing activity at the network level as well. Transcranial magnetic stimulation can synchronize the firing of groups of neurons, causing them to work more cohesively within specific brain networks. This synchronized activity is believed to be crucial for various cognitive functions (Wagner et al., 2009).

Transcranial magnetic stimulation works by inducing noninvasively electric currents in localized cortical regions, thus modulating their excitability levels and ongoing activity patterns depending on stimulation settings: frequency, number of pulses, train duration, and intertrain intervals. Distinct types of TMS have been devised depending on the frequency and type of magnetic pulse delivered. Single-pulse TMS discharges a single magnetic pulse at a given time, whereas repetitive pulse TMS (rTMS) delivers a repeated single magnetic pulse of the same intensity. Theta burst stimulation (TBS) is a novel paradigm consisting of short bursts at 50–100 Hz stimulation frequency that is repeated at 5 Hz (“theta frequency”). This protocol can be intermittent (iTBS) or continuous (cTBS). For the past years, different protocols of TMS for different disorders, such as obsessive-compulsive disorder (OCD), addiction, or MDD have been developed to find better treatments than the existing ones. As a result, the FDA has cleared a TMS protocol for the treatment of MDD and TRD in adult population (Janicak et al., 2008; Croarkin et al., 2010; Serafini et al., 2015).

Transcranial magnetic stimulation treatment for cognitive deficit is entering a new developmental and sophistication phase. Previous studies have associated TMS with significant improvement in some cognitive performance (Ilieva et al., 2018; Schaffer et al., 2020). However, others could not confirm the cognitive-enhancing properties of TMS (Holtzheimer et al., 2004; Wajdik et al., 2014). Still, there is insufficient knowledge of the differential effects of TMS protocols and stimulation parameters on cognitive control in MDD. Therefore, this review aims to synthesize the effectiveness of the TMS methods and a qualitative assessment of their potential benefits in improving cognitive functioning in patients with MDD.

## Methods

We conducted a systematic review to synthesize the evidence that different TMS protocols provide regarding cognitive control in patients diagnosed with MDD. This review followed a predefined protocol registered in PROSPERO (https://www.crd.york.ac.uk/prospero/display_record.php?ID=CRD42022379709.

**Fig. 1:**
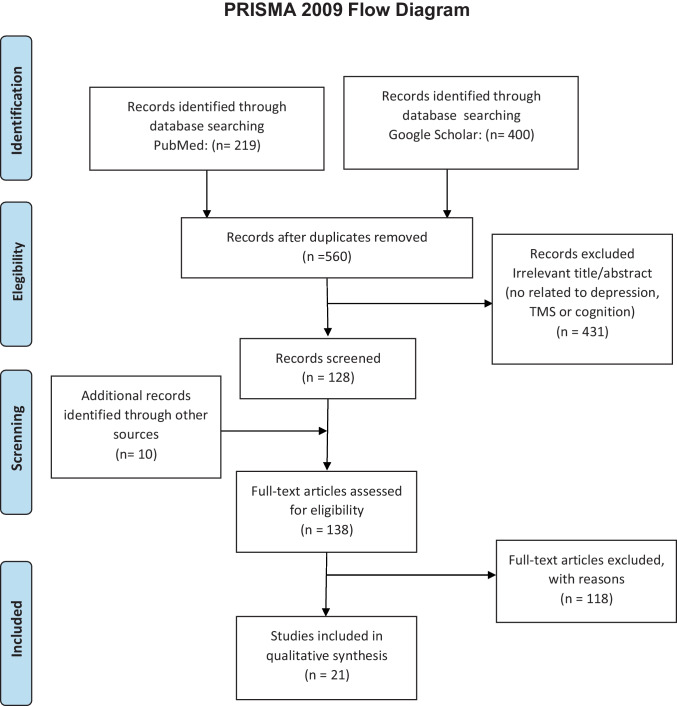
PRISMA flow diagram, illustrating the identification and selection of relevant studies in the systematic review

The guidelines and recommendations in the PRISMA statement (Page et al., 2021) have been followed to structure the gathered information reliably in this systematic review (Fig. 1).

### Information sources and search

Study selection consisted of two different steps. First, we used standard electronic databases: Google Scholar (https://scholar.google.com/) and PubMed (https://pubmed.ncbi.nlm.nih.gov/) to identify articles using different protocols of TMS in cognitive control in depressed patients. We only reported the results from these two databases, because results from other sources (e.g., Medline or Web of Sciences) did not provide any result of our interest. Second, we examined the references of review studies to identify other relevant articles. We searched for any paper published in English, French, or Spanish, but only English papers were selected. The first studies relevant to our search were from 2002, and the most recent was from 2023.

The systematic search performed in the databases was done by using a series of keywords. First, we focused our attention on the technique, using words, such as “noninvasive brain stimulation,” “transcranial magnetic stimulation,” “TMS treatment,” and “Theta burst stimulation.” Second, keywords were used to identify articles that studied cognitive control: “cognitive function”; “cognitive deficit”; “cognitive effect”; “neurocognition”; “cognitive performance”; and “cognitive deficit.” These searches were always accompanied by terms, such as “depressed patients,” “major depressive disorder,” “treatment-resistant depression, and “depression” to refer to the pathology. When a title or abstract seemed to describe a study eligible for inclusion, the full-text article was examined to assess its relevance based on the inclusion criteria.

### Eligibility Criteria

Only studies focused on depressed patients were included in this review. Other pathologies and comorbidities were excluded.

We have opted to exclude patients younger than 18 years because of ongoing concerns regarding the safety and effectiveness of TMS for adolescents. While TMS is a noninvasive procedure, its safety profile in this age group remains under investigation. For example, while Qiu et al. (2023) suggests potential benefits in a systematic review, a large-scale randomized controlled trial (RCT) designed to expand FDA clearance for rTMS yielded negative results (Oberman et al., 2021). Specifically, no significant difference was observed between the rTMS and sham control groups. Given these uncertainties, along with the ethical considerations of exposing minors to experimental treatments, excluding them minimizes potential risks. Moreover, considering that the neural networks involved in a developing brain might differ, we have chosen to exclude these investigations from the analysis to enhance clarity in interpreting the results (Haan & Johnson, 2005).

In addition, we discarded observational and review studies. The protocol of the selected studies included any transcranial magnetic stimulation, such as repetitive TMS, low- frequency TMS, and intermittent and continuous theta-burst stimulation.

Moreover, the primary or secondary outcome measures were any change in cognitive control processes, including verbal and visuospatial memory, inhibition, sustained attention, working memory, set-shifting, cognitive flexibility, abstract reasoning, psychomotor speed, phonemic fluency, etc.

### Data collection process

All relevant data were extracted and processed in an Excel spreadsheet. Selected information was categorized according to (1) characteristics of the sample: sample size, mean age and SD, and gender; (2) depression information: type of depression diagnosed and pharmacological treatment (Table 1); (3) TMS information: type of TMS and type of coil; (4) TMS protocol: frequency, intensity, duration of protocol, pulses per session; (5) Localization: stimulation site and localization method; (6) control condition: size of control group, control stimulation; (7) study design; (8) task performed and cognitive domain; and (9) TMS effect (Table 2 and 3).

**Table 1:** Bibliographic and demographic characteristics of the studies included in the review

Study	Sample total N	Mean age (SD or range)	Sex	Medication	Type of depression/treatment
Bahun et al. (2022)	32 total 2 weeks: 18 3 weeks: 14	2 weeks: 51, 72 (11, 1) 3 weeks: 56 (10, 81)	2 weeks: 8 M; 10 F 3 weeks: 8 M; 6 F	At least 1 antidepressant (11 participants were taking 2 antidepressants)	Treatment-resistant major depression
Bermpohl et al. (2006)	18 total Depressed: 10 Improved: 8	Depressed: 56, 8 (9, 3) Improved: 50, 6 (9, 4)	Depressed: 4 M; 6 F Improved: 3 M; 5 F	At least 1 antidepressant	Major depression
Cheng et al. (2016)	60	NS (21–70)	NS	At least 1 antidepressant	Medication-resistant depression
Concerto et al. (2015)	30 total Test: 15 Control: 15	Test: 51 (6, 5) Control: 53 (6, 7)	Test: 9 M; 6 F Control: 8 M; 7 F	On their original medication	Treatment-resistant depression
Corlier et al. (2020)	71 total Responders: 23 Nonresponders: 48	Responders: 43, 4 (16, 7) Nonresponders: 42, 6 (14, 5)	Responders: 34.8% M Nonresponders: 41.7% M	60.9% antidepressant	Treatment-resistant depression
Holczer et al. (2021)	20	50, 27 (13, 24)	5 M; 15 F	Benzodiazepine and/or antidepressant	Unipolar major depression
Kavanaugh et al. (2018)	84 total Sham: 41 Active: 43	Sham: 47, 95 (12, 78) Active: 45, 86 (11, 57)	Sham: 10 M; 31 F Active: 12 M; 31 F	No psychotropic medication at all, or on a stable psychotropic regimen for at least 6 weeks before baseline	Treatment-resistant depression
Kedzior et al. (2012)	18 total Patients: 10 Volunteers: 8	Patients: 43 (9) Volunteers: 39 (15)	Patients: 5 M; 5 F Volunteers: 6 M; 2 F	Pharmacotherapy stable for 2 weeks	Treatment-resistant depression
Kuroda et al. (2006)	23 total Patients: 9 Controls: 14	Patients: 36, 4 (6, 1) Controls: 34, 9 (8, 7)	Patients: 4 M; 5 F Control: M 2; 12 F	Fluvoxamine and Lorazepam	Treatment-resistant depression
Levkovitz et al. (2009)	84 total H1-120%: 23 H2-120%: 22 H1L-120%: 11 H1L-110%: 8 Control: 20	H1-120%: 45, 57 (13, 34) H2-120%: 45, 77 (11, 99) H1L-120%: 44, 27 (11, 36) H1L-110%: 49, 88 (9, 52) Control: 43, 90 (18, 21)	H1-120%: 12M; 11F H2-120%: 11M; 11F H1L-120%: 4M; 7F H1L-110%: 3M; 5F Control: 9M; 11F	Medication-free	Unipolar treatment-resistant depression
Martis et al. (2003)	15	43, 5 (15, 8)	12 M; 3 F	Medication-free	Treatment-resistant depression
Moser et al. (2002)	19 total Active: 9 Sham: 10	Active: 61, 22 (10, 3) Sham: 60, 90 (10, 2)	NS	Medication reduction	Treatment-resistant depression
Nadeau et al. (2014)	48 total R rTMS: 16 L rTMS: 18 R Sham: 7 L Sham: 7	R rTMS: 48, 5 (10, 8) L rTMS: 46,7 (15, 3) R Sham: 46, 6 (20, 2) L Sham: 41, 9 (14, 1)	R rTMS: 7M; 9F L rTMS: 4M; 14F R Sham: 3M; 4F L Sham: 5M; 2 F	At least 1 antidepressant	Treatment-resistant depression
Naim-Feil et al. (2016)	47 total Patients: 21 Healthy: 26	Patients: 44 (9) Healthy: 39 (12)	Patients: 10 M; 11 F Healthy: 15M; 11F	At least 1 antidepressant	Treatment-resistant depression
Pallanti et al. (2012)	28	41, 3 (8, 3)	12 M; 16 F	At least 1 antidepressant (SSRI)	Treatment-resistant depression
Rostami et al. (2022)	120 total UDD: 56 BDD: 64	33, 58 (11, 1)	49 M; 71 F	NS	Major depression (unipolar and bipolar)
Şalçini et al. (2018)	39 total Patients: 19 Healthy: 20	Patients: 35, 95 (10, 24) Healthy: 32, 47 (6, 35)	Patients: 6 M; 13 F Healthy: 9 M; 11 F	Medication-free	Treatment-resistant depression
Schaffer et al. (2020)	53	43, 38 (14, 32)	19 M; 34 F	At least 1 antidepressant	Treatment-resistant depression
Spampinato et al. (2013)	22 total Test: 12 Control: 10	Test: 50, 50 (8, 69) Control: 55, 60 (8, 78)	Test: 8 M; 4 F Control: 6 M: 4 F	At least 3 different antidepressant	Treatment-resistant depression
Vanderhasselt et al. (2009)	16	42 (11, 2)	6 M; 10 F	Medication free	Major depression
Yildiz et al. (2023)	45 total Patients-active: 15 Patients-sham: 15 Healthy control: 15	Patients-active: 40, 60 (7, 21) Patients-sham: 37, 73 (9, 33) Healthy control: 38, 40 (8, 5)	Patients-active: 2 M; 13 F Patients-sham: 6 M; 9 F Healthy control: 5 M; 10 F	NS	Major depression

**Table 2:** Summary of reviewed TMS studies, including stimulation parameters and results

Study	TMS protocol	Type of coil	Frequenc y of sessions	Intensity	Duration	Pulses per sessio n	Stimulatio n site	Localizatio n method	Group Active/Sha m	Study design	Cognitive domain	Task	TMS effect
Bahun et al. (2022)	dTMS	H1 coil devic e	18 Hz, twice a day	120% of motor threshold	20 min session; 2 weeks (10 working days) 3 weeks (15 working days)	1980	Left DLPFC Control condition: none	NS	None	Randomize d trial	Verbal memory, Numeric memory, visual memory, visuospatial and executive function, phonemic fluency	Auditory-verbal learning test (AVLT), Verbal- logic memory, Digit span task, Benton visual retention test (BVRT), Trail Making test (TMT), Block design subtest, FAS Verbal fluency test	**2-week dTMS and 3-week dTMS:** ↑ cognition on cognitive all tasks **3**-**week dTMS:** ↑DSST performance vs 2-week dTMS
Bermpohl et al. (2006)	LF-rTMS	Figure -of- eight coil	1 Hz, once	60% of maximum stimulator output	10 min session; 1 day	NS	Left DLPFC; right DLPFC Control condition: mesial occipital cortex (active control)	Moving the coil 5 cm anteriorly from the point of MT determinatio n	Active control same group as test	Pseudo- randomized controlled study	Inhibition	Affective go/no- go task	**Right Prefrontal rTMS**: ↑ performance in depressed patients **Left Prefrontal rTMS:** ↓ performance in improved group •No effect on depressed patients
Cheng et al. (2016)	iTBS; cTBS	Figure-of- eight coil	5 Hz daily	80% active MT	5 consecutiv e days with a total of 10 sessions during 2 weeks	A: 1800 B: 1800 C: 1800 + 1800 =36000 D (sham): 1800	Left DLPFC; right DLPFC Control condition: Sham TBS coil 90º to the skull	MRI and brain navigation	45/15	Randomize d double- blind sham- controlled study	Attention, perseverance, WM, abstract thinking, and set shifting Executive function in general	Wisconsin Card Sorting test (WCST)	**Left Prefrontal iTBS:** •significant improvement on EF Cognition- enhancing effect was independent from the antidepressant effect
Concerto et al. (2015)	HF - rTMS	Figure -of- eight coil	10 Hz, daily	120% resting MT	5 days per week for 4 weeks	3000 (37,5 min)	Left DLPFC Control condition: coil at 45º touching but not stimulating the skull	Moving the coil 5 cm anteriorly from the point of MT determinatio n	15/15	Randomize d controlled study	Abstract reasoning, flexibility, inhibition,	Frontal assessment battery (FAB), Stroop T	**Left DLPFC HF- rTMS:** ↓ depression rating scale scores lasting for 6 months ↑ STROOP T (transient)
Corlier et al. (2020)	rTMS	Figure -of- eight coil	10 Hz	120% MT	30 sessions	NS	Left DLPFC Control condition: None	Beam F3 method	None	Quasi- experimental design with a single group	Cognitive flexibility and attention span, inhibition	Stroop T	**Left DLPFC rTMS:** ↓ depression symptoms ↑ accuracy and reaction times STROOP = benefit in inhibition and flexibility ↑ accuracy better for older patients in incongruent conditon
Holczer et al. (2021)	cTBS; iTBS	Figure -of- eight coil	50 Hz, daily	30% of the maximal stimulator output for all participant s Resting MT	10 workdays	cTBS: 600 pulses iTBS: 600 pulses	left DLPFC; right DLPFC Control condition: plastic block elevated the coil from the scalp by 4 cm	MRI and brain navigation	10/10	Randomize d sham- controlled study	Working memory, attention	N back task, attention network task (ANT)	**Bilateral DLPFC TBS:** ↑ affective symptoms •No effects on WM and attentional domains
Kavanaugh et al. (2018)	rTMS	Two coil devic e	10 Hz, daily	Less than or equal to 120% of the participant' s left-sided resting motor threshold	20 sessions for 4 to 6 weeks	3000	Left DLPFC and bilateral dorsomedi al prefrontal cortex (midline) Control condition: coil same position but no energy delivered	Anatomical landmark	43/41	Randomize d, double- blind, sham- controlled trial	Simple reaction time, choice reaction time, digit vigilance, numeric working memory, spatial working memory, immediate and delayed word recall, word recognition, and picture recognition	Word and picture presentation and recognition, digit vigilance task, spatial working memory, numeric working memory, delayed recall	**2-coils rTMS left DLPFC and Dorsomedial PFC:** ↑ quality of episodic memory •No effects for attention or WM
Kedzior et al. (2012)	Fast frequenc y rTMS	NS	10 Hz, daily	100% motor threshold	20 sessions over 20 consecutiv e working days	2000	Left DLPFC Control condition: no stimulation to healthy volunteers just cognitive task	10-20 system	10/8 healthy volunteers	Pilot, one treatment- group only design	Immediate memory, visuospatial/constructional ability, language, attention, and delayed memory	Modified Concept-Shifting Task (mCST), Repeatable Battery for the Assessment of Neuropsychologic al Status (RBANS),	**Left DLPFC ff- rTMS:** ↓ depression severity ↑ concept-shifting ability and immediate memory
Kuroda et al. (2006)	rTMS	Figure-of- eight coil	10 Hz, daily	100% motor threshold	10 sessions, 5 times per week for 2 consecutiv e weeks	1000	Left DLPFC Control condition: no stimulation to healthy volunteers just cognitive task	Moving the coil 5 cm anteriorly from the point of MT determinatio n	9/14	Open trial lacking sham- treated controls	Visual immediate, verbal immediate, delayed memory function, psychomotor speed, sequencing, visual scanning	Mini-mental State Examination (MMSE), Wechsler Memory Scale Revised (WMSR), TMT,	**Left DLPFC rTMS:** ↓ Hamilton Rating Scale for Depression ↑verbal memory
Levkovitz et al. (2009)	dTMS	H1, H2, H1L	20 Hz	110 or 120% of MT	20 sessions, 5 times per week for 4 consecutiv e weeks	1680	Left DLPFC Control condition: no stimulation to healthy volunteers just cognitive task	Moving the coil a little more anterior than 5,5 cm from the point of MT determinatio n	65/20	Randomize d controlled study	Sustained attention, visuospatial memory, executive functions, psychomotor speed	Rapid visual processing (RVP), Paired associates learning (PAL), Stockings of Cambridges (SOC), spatial working memory (SWM), Reaction time task (RTI),	**Left DLPFC HF dTMS:** ↓ depression symptoms ↑ cognition, especially for H1 and H1L-120% groups
Martis et al. (2003)	rTMS	Figure-of- eight coil	10 Hz, daily	110% MT	10-20 sessions over 2-4 weeks	NS	Left prefrontal stimulation Control condition: None	Moving the coil 5 cm anteriorly from the point of MT determinatio n	None	Open randomize d study	Speed of information processing, inhibition, speeded word retriveal, working memory,anterograde memory for verbal and visual information	Simple and choice reaction time, stroop test, verbal fluency, WAIS III letter number span, WMSR visual reproduction and logical memory	**Left PFC rTMS:** ↑ WM, Executive function, objective memory, fine motor speed domains • Better HDRS scores
Moser et al. (2002)	rTMS	Figure -of- eight coil	20 Hz, daily	80% MT	5 sessions	800	Anterior portion of the left middle frontal gyrus Control condition: same stimulation parameter s but with the figure eight coil above the top of the skull with the handle placed against the head	MRI and brain navigation	9/10	Randomize d sham- controlled study	Executive function, language, memory, visuospatial	Trail Making test–A and –B, Stroop test, WAIS-R Digit symbol; Controlled oral word association; Boston naming test, Sentence Repetition; Rey Auditory Verbal Learning Test— % of learned words recalled after delay; Judgment of Line Orientation.	**Anterior portion of the left middle frontal gyrus rTMS**: ↑Trial-Making Test B performance
Nadeau et al. (2014)	rTMS	Figure -of- eight coil	5 Hz, daily	100% MT	10 weekday sessions for 2 weeks	2000	Left DLPFC; right DLPFC control condition: Magstim Placebo Coil System + electrical stimulation with electrodes	Moving the coil 5 cm anteriorly from the point of MT determinatio n	16 R 18 L/14	Randomize d sham- controlled study	Language, visuospatial function, executive function, verbal episodic memory, attention	Boston Naming test, category fluency, Block design subtest, Stroop test, TMT, Oral word association test letter fluency, California verbal learning test, Paced auditory serial addition test	**Right DLPFC rTMS:** ↑attention, language function, visuospatial function, and episodic memory compared to left DLPFC rTMS
Naim-Feil et al. (2016)	dTMS	H1 coil	20 Hz, daily	120% MT	20 sessions, 5 times per week for 4 consecutiv e weeks	1680	Left DLPFC control condition: no stimulation to healthy Volunteers just	Moving the coil 6 cm anteriorly from the point of MT determinatio n	21/26 Healthy subjects	Quasi- experimental design with a nonequivalent control group	Working memory, sustained attention, and impulse/inhibitory control	Sustained attention to responde task (SART)	**Acute and long- term left DLPFC HF-dTMS:** ↑ sustained attention
Pallanti et al. (2012)	LF-rTMS	Figure -of- eight coil	1 Hz, daily	110% resting MT	15 sessions on weekdays	420	right DLPFC control condition: None	5 cm anterior to the stimulation site for the contralateral abductor pollicis brevis in the parasagittal plane	None	Randomize d study lacking sham- treated controls	Visual short-term memory, visuospatial learning, verbal fluency	Corsi block- tapping test, phonemic verbal fluency	**Right DLPFC lf- rTMS:** ↑ performance in corsi block tapping test ↑phonemic verbal fluency (not significant) ↓ HAM-D score
Rostami et al. (2022)	HF- rTMS LF- rTMS	Air- cooled figure-of-8 coil	left: 10 Hz, daily right: 1 Hz, daily	left: 110% resting MT right: 120% resting MT	20 sessions	Left: 3750 right: 1500	Left DLPFC; right DLPFC control condition: None	NS	None	Retrospectiv e naturalistic study	Sustained attention, working memory, recognition, spatial planning	Spatial working memory, delayed matching to sample, one touch stockings of Cambridge,	**Bilateral DLPFC rTMS:** ↑ cognitive function, sustained attention, WM, and executive functions ↑ WM in UDD patients vs BDD patients ↓depressive symptoms in UDD and BDD
Şalçini et al. (2018)	rTMS	Figure -of- eight coil	25 Hz, daily	100 % MT	20 consecutiv e days (except Sunday)	1000	Left DLPFC control condition: sham coil using the same procedure as the patient group	Moving the coil 5 cm anteriorly from the point of MT determinatio n	19/20 healthy volunteers	Sham- controlled study	Inhibition, attention, visuospatial function, flexibility	Stroop test, TMT, WCST	**Left DLPFC rTMS:** ↓depressive symptoms ↑ cognitive functions (executive functions)
Schaffer et al. (2020)	LF TMS	Figure -of- eight coil	1 Hz, daily	100% resting MT	6 weeks	1200	Right DLPFC and SMA control condition: none	Moving the coil 5 cm anteriorly from the point of MT determinatio n	None	Nonrandomized study	Neurocognition index, composite memory, verbal memory, visual memory, processing speed, executive function, psychomotor speed, reaction time, complex attention, cognitive flexibility	Visual memory test, verbal memory test, finger tapping, symbol digit coding, stroop test, shifting attention test, continuous performance test	**Right DLPFC and SMA lf- TMS:** ↓ depressive symptoms ↑ neurocognition index, executive function and cognitive flexibility
Spampinato et al. (2013)	HF - rTMS	Figure -of- eight coil	10 Hz, daily	130% resting MT	20 sessions, 5 times per week for 4 consecutive weeks	3000	Left DLPFC control condition: coil at 45 to touch but not stimulate the skull	Moving the coil 5 cm anteriorly from the point of MT determination	12/10	Randomize d sham- controlled study	Abstract reasoning, flexibility, inhibition	Frontal assessment battery (FAB), Stroop T	**Left DLPFC HF- rTMS:** ↑ neuropsychological test scores ↑ STROOP test but not in FAB
Vanderhasselt et al. (2009)	HF - rTMS	Figure-of-eight coil	10 Hz,onces	110% MT	1 single session	1560	Left DLPFC control condition: coil at 45 to touch but not stimulate the skull.	MRI and brain navigation	Sham same group as real but with 1 week difference	Double- blind, placebo- controlled crossover	Attention, inhibition	Switching task	**Left DLPFC HF- rTMS:** ↑performance in switching task
Yildiz et al. (2023)	rTMS	Figure-of-eight coil	10 Hz, daily	110% MT	20 session	1000	Left DLPFC control condition: coil at 45 to touch but not stimulate the skull	Moving the coil 5 cm anteriorly from the point of MT determinatio n	15/15	Randomized sham- controlled study	Inhibition, attention, visuospatial function, flexibility, memory	WCST, Stroop test, TMT, Digit Span test (DST), Verbal memory process test (VMPT)	**Left DLPFC rTMS:** ↑ performance in Stroop test and and WCST

**Table 3:** Results of reviewed studies organized by cognitive domain

Cognitive domain	Study	Test	Detail result
**Visuospatial function and recognition**	Nadeau et al. (2014)	Block design subtest	Baseline vs. 3 months: - visuospatial function: not significant (*p* = 0.013)
	Bahun et al. (2022)	Block design subtest	Main effect of time: significant (*p* = 0.022) Main effect of modality (2 w vs. 3 w): Not significant (*p* = 0.203)
	Rostami et al. (2022)	One touch stockings of cambridge	Pre vs Post: - Significant
	Levkovitz et al. (2009)	Stockings of Cambridges (SOC)	Baseline vs. visit 21 (5 w): - H1: significant - H1L-120%: significant
	Moser et al. (2002)	Judgment of line orientation	Baseline vs. follow-up:’ - Not significant difference
	Kedzior et al. (2012)	Repeatable battery for the assessment of neuropsychological status (RBANS)	Before rTMS vs. after (20 days): - Not significant (*p* = 0.083)
**Executive function**	Bahun et al. (2022)	Trail Making test	Main effect of time TMT-time: significant (*p* = 0.022) Main effect of time TMT-errors: not significant (*p* = 0.705) Main effect of modality TMT-time (2 w vs. 3 w): not significant (*p* = 592) Main effect of modality TMT-errors (2 w vs. 3 w): not significant: (*p* = 0.657)
	Kuroda et al. (2006)	Trail Making test	Session 0 vs. Session 10: - Not significant
	Moser et al. (2002)	Trail Making test	Baseline vs. follow-up: - TMT-B: significant (*p* < 0.05)
		WAIS-R Digit symbol	Baseline vs. follow-up: - Not significant difference
	Nadeau et al. (2014)	Trail Making test	Baseline vs. 3 months:’ - Executive function: not significant (*p* = 0.944)
	Yildiz et al. (2023)	Trail Making test	Before vs. after rTMS: - Significant (TMTA *p* = 0.001;TMTB *p* = 0.011) Active vs. sham: - Not significant (TMTA *p* = 0.704;TMTB *p* = 0.632)
	Şalçini et al. (2018)	Trail Making test	Session 0 vs. session 20: - Not significant (*p* > 0.05)
	Concerto et al. (2015)	Frontal assessment battery (FAB)	Before vs. after rTMS: - Not significant
	Spampinato et al. (2013)	Frontal assessment battery (FAB)	Before vs. after (4w) rTMS: - Not significant (*p* = 0.110)
**Phonemic fluency**	Pallanti et al. (2012)	FAS verbal fluency test	Baseline vs. 3w: - Patients: Not significant (*p* = 0.065) - Responders: Not significant (*p* = 0.65) - Nonresponders: Not significant (*p* = 0.43)
	Bahun et al. (2022)	FAS verbal fluency test	Main effect of time: no significant (*p* = 0.085) Main effect of modality: no significant (*p* = 0.988)
	Martis et al. (2003)	Oral word association test	Baseline vs. post rTMS: - Working memory and executive function: Significant improvement (*p* = 0.01)
	Nadeau et al. (2014)	Boston naming test	Baseline vs. 3 months: - Language: Not significant (*p* = 0.579)
	Moser et al. (2002)	Boston naming test	Baseline vs. follow-up: - Not significant difference
		Sentence repetition	Baseline vs. follow-up: - Not significant difference
**Inhibition**	Bermpohl et al. (2006)	Affective go/no-go task	Right rTMS vs. control: - Depressed: Significant reduced error number (*p* = 0.02) Improved: Not significant Left rTMS vs. control - Depressed: Not significant Improved: Significant increase error number (*p* = 0.05)
	Concerto et al. (2015)	Stroop test	T0 vs. T1 (end of rTMS): Significant (*p* = 0.0013) T0 vs. T2 (3 months after rTMS): Not significant T0 vs. T3 (6 months after rTMS): Not significant
	Corlier et al. (2020)	Stroop test	Pre vs. post rTMS: - Responders: Incongruent condition: significant
	Martis et al. (2003)	Stroop test	Baseline vs. post rTMS: - Attention and mental speed: Not significant improvement (*p* = 0.58)
	Moser et al. (2002)	Stroop test	Baseline vs. follow-up: - Not significant difference
	Nadeau et al. (2014)	Stroop test	Baseline vs. 3 months: - Executive function: Not significant (*p* = 0.944)
	Şalçini et al. (2018)	Stroop test	Baseline vs. follow-up (20 sessions): - Significant (*p* < 0.05)
	Schaffer et al. (2020)	Stroop test	Pre vs. Post rTMS: - Reaction time: Not significant - Complex attention: Significant (*p* < 0.01) - Cognitive flexibility: Significant (*p* < 0.001)
	Yildiz et al. (2023)	Stroop test	Active before vs active after rTMS: - Significant in all three categories (*p* = 0.008; 0.003; 0.012) Active vs. sham: - Significant neutral words (*p* = 0.045)
	Spampinato et al. (2013)	Stroop test	Baseline vs. after treatment: - Signifcant (*p* = 0.00004)
**Attention**	Holczer et al. (2021)	Attention network task (ANT)	Effect time x stimulation: - Not significant (*p* = 0.161)
	Kavanaugh et al. (2018)	Digit vigilance task	Time by treatment: - Continuity of attention: Not significant (*p* = 0.401) · Responders: Not significant (*p* = 0.970) · Remitter: Not significant (*p* = 0.404) - Power of attention: Not significant (*p* = 513) · Responders: Not significant (*p* = 0.513) · Remitter: Not signifcant (*p* = 0.902)
	Levkovitz et al. (2009)	Rapid visual processing (RVP)	Baseline vs. visit 21 (5 w): H1: signifcant H2: significant H1L-110%: Not significant H1L-120%: Significant
	Naim-Feil et al. (2016)	Sustained attention to response task (SART)	Baseline vs. single session: significant (*p* = 0.036) Baseline vs. long-term treatment: significant (*p* = 0.023)
	Schaffer et al. (2020)	Continuous performance test	Pre vs. Post rTMS: - Complex attention: Significant (*p* < 0.01)
	Kedzior et al. (2012)	Repeatable battery for the assessment of neuropsychological status (RBANS)	Before rTMS vs. after (20 days): - Not significant (*p* = 0.150)
	Martis et al. (2003)	Squire test and simple and choice reaction time	Baseline vs post rTMS: - Attention and mental speed: Not significant improvement (*p* = 0.58)
	Nadeau et al. (2014)	Paced Auditory serial addition test	Baseline vs. 3 months: - Attention: Significant (*p* < 0.0001)
**Set shifting/flexibility**	Cheng et al. (2016)	Wisconsin card sorting test (WCST)	Baseline vs. Post TBS: - iTBS responders: significant · total corrects (*p* = 0.027) · total errors (*p* = 0.027) · % errors (*p* = 0.028) · % conceptual level responses (*p* = 0.028) · Completed categories (*p* = 1.0)
	Yildiz et al. (2023)	Wisconsin card sorting test (WCST)	Before vs. after rTMS: - Significant: nb of categories completed (*p* = 0.017); total nb of correct responses (*p* = 0.046); total nb of false responses (*p* = 0.046) Active vs. sham: - Not significant
	Kedzior et al. (2012)	Modified Concept-Shifting Task (mCST)	Before vs. After rTMS (Day 20): - Duration: significant (*p* = 0.047) - Accuracy: significant (*p* = 0.038)
	Vanderhasselt et al. (2009)	Switching task	Pre vs Post treatment: - Auditory switch trials: significant (*p* < 0.05) - Visual switch trials (DT): significant (*p* < 0.05) - Visual switch trials (MT) not significant
**Working memory**	Holczer et al. (2021)	N back task	Time x stimulation: - 2 back and 3 back task: No significant (*p* = 0.249)
	Kavanaugh et al. (2018)	Spatial Working Memory (SWM)	Time by treatment: - Quality of working memory: Not significant (*p* = 0.294) · Responders: Not significant (*p* = 0.635) · Remitter: Not significant (*p* = 0.708)
		Numeric Working Memory	-
	Levkovitz et al. (2009)	Spatial Working Memory (SWM)	Baseline vs. visit 21 (5 w): - H1: significant - H2: significant - H1L-120%: significant
	Martis et al. (2003)	WAIS III: Letter number span	Baseline vs. post rTMS: - Working memory-executive function: Significant (*p* = 0.01)
	Rostami et al. (2022)	Spatial Working Memory (SWM)	Pre vs. Post treatment: - Significant
		delayed matching to sample	Pre vs. Post: - Significant
	Yildiz et al. (2023)	Digit span task	Before vs. after rTMS: - Not significant (*p* = 0.157) Active vs. sham: - Not significant (*p* = 0.539)
	Bahun et al. (2022)	Digit span task	-
**Memory** **(Verbal and visual memory included)**	Kavanaugh et al. (2018)	Delayed Word Recall	Time by treatment: - Quality of episodic memory: Significant (*p* = 0.028) · Responders: Not significant (*p* = 0.231) · Remitter: Not significant (*p* = 0.0.110)
		Word and picture presentation and recognition	-
	Kuroda et al. (2006)	Wechsler Memory Scale Revised (WMSR)	Before vs. after rTMS: - paired words/unrelated/immediate: significant - paired word/unrelated/delayed: significant
		Everyday Memory Checklist (EMC)	Before vs. after rTMS: - Significant
	Martis et al. (2003)	Wechsler Memory Scale Revised (WMSR)	Baseline vs. post rTMS: - Objective Memory: Significant (*p* < 0.01)
	Kedzior et al. (2012)	Repeatble battery for the assessment of neuropsychological status (RBANS)	Before vs After rTMS (Day 20): - Immediate memory: Significant (*p* = 0.030)
	Pallanti et al. (2012)	Corsi block Tapping test	Baseline vs. 3 w: - Patients: signifcant (*p* = 0.011) - Responders: Not significant (*p* = 0.087) - Nonresponders: Not significant (*p* = 0.14)
	Nadeau et al. (2014)	California verbal learning test	Baseline vs. 3 months: - Verbal memory: Not significant (*p* = 0.005)
	Bahun et al. (2022)	Auditory-verbal learning test	Main effect of time: significant (*p* = 0.000) Main effect of modality (2 w vs. 3 w): Not significant (*p* = 0.380)
		verbal logic memory	Main effect of time: significant (*p* = 0.015) Main effect of modality (2 w vs. 3 w): Not significant (*p* = 0.760)
		Benton visual retention test (BVRT)	BVRT correct: Main effect of time: significant (*p* = 0.015) Main effect of modality (2 w vs. 3 w): Not significant (*p* = 0.113)
	Moser et al. (2002)	Rey auditory verbal learning test	Baseline vs. follow-up: - Not significant difference
	Levkovitz et al. (2009)	Paired associates learning (PAL)	Baseline vs. visit 21 (5 w): - H1: significant
	Schaffer et al. (2020)	Visual memory test	Pre vs. Post rTMS: - Visual memory: Not significant
	Yildiz et al. (2023)	Verbal memory process test (VMPT)	Before vs. after rTMS: - Not significant Active vs. sham: - Not significant
**Speed of information processing and motor functioning**	Levkovitz et al. (2009)	Reaction Time task (RTI)	Baseline vs. visit 21 (5 w): - Not significant
	Martis et al. (2003)	Simple and Choice reaction time	Baseline vs. post rTMS: - Attention and mental speed: Not significant (*p* = 0.58)
	Schaffer et al. (2020)	Finger tapping	Pre vs Post rTMS: - Psychomotor speed: Significant (*p* < 0.05)

The data synthesis was planned in a narrative manner, including various table overviews. We focused on a qualitative synthesis to describe the studies’ main contributions, outstanding findings, applicability, and limitations, due to the high variability in the cognitive tasks and TMS protocols used. The synthesis was structured around the stimulation protocol, the cognitive tests, and the predominant cognitive effect.

To understand the effects of stimulation in the brain areas, we analysed the behaviour and results in the different cognitive tasks and interpreted their outcomes based on existing literature. These outcomes were measured with different variables, such as reaction time, accuracy, and congruent effects among others. These tests reported post-treatment results (mean, SD, *p*-value, Z, T, chi-squared).

## Results

### TMS protocol

All of the studies included in this review used Transcranial Magnetic Stimulation to change the brain’s cortical excitability. Each study used different TMS protocols depending on the pulse’s delivery and the frequency (low or high): three studies used deep TMS (dTMS); two studies used continuous TBS (cTBS) and intermittent TBS (iTBS); four used low-frequency repetitive TMS (LF-rTMS); five used high-frequency (or fast) repetitive TMS (HF-rTMS); and seven used repetitive TMS (rTMS).

### Repetitive TMS

rTMS refers to any combination of three or more pulses of a specific intensity delivered at a frequency of at least 0.5 pulses per second. In contrast to the transient effects of single- and paired-pulse TMS, longer trains of rTMS have been shown to induce more sustained activity and metabolic changes than the stimulation itself (Burke et al., 2019).

rTMS has two classic paradigms: LF-rTMS consisting of one continuous train of impulses at 1 Hz or less, and HF-rTMS consisting of stimuli typically lasting 5–10 s at 5 Hz or higher and separated by 20–50-s pauses.

Protocol by Kavanaugh et al. (2018), Kuroda et al. (2006), and Yildiz et al. (2023) consisted of 20 trains of 5-s duration for each session, followed by a 26-s and 25-s pause, respectively. The stimulation for both protocols was applied at a 10-Hz frequency. Martis et al. (2003) used the same frequency, but the trains lasted 5 s with 30 s of intertrain intervals. Other studies had applied higher frequencies, such as Moser et al. (2002), whose subjects received 5 sessions of 20-Hz stimulation for 2 s with 20 trains separated by 1-min pauses, or Şalçini et al. (2018) whose stimulation consisted of 20 pulse trains of 2 s at 25 Hz and separated by 30-s intertrain intervals. Finally, Nadeau et al. (2014) used a daily session of 2000 pulses divided into 50 trains of 40 stimuli. Each train lasted 8 s and the between train was 22 s.

From the studies included, three used LF-rTMS, and all of them used 1-Hz frequency (Bermpohl et al., 2006; Pallanti et al., 2012; Schaffer et al., 2020; Rostami et al., 2022). Pallanti et al. (2012) used a three 140-s train at 1 Hz with a 30-s intertrain interval. These parameters are now considered widely safe as they followed Rossi et al. (2009) as a safety consensus. Schaffer et al. (2020), however, used 1200 pulses at 1 Hz with stimulation delivered in 1-s pulses. Rostami et al. (2022) included a 10-s train of 1 Hz, with an intertrain interval of 2 s.

Regarding HF-rTMS, all studies selected for this review set the intensity at 10 Hz. Concerto et al. (2015) and Spampinato et al. (2013) used the same protocol of 4-s train duration and 26-s intertrain interval, for a total of 3000 pulses per session lasting 37.5 min. Kedzior et al. (2012) and Rostami et al. (2022) set the trains at 5 s, but the intertrain was different, lasting 25 and 10 s, respectively. Vanderhasselt et al. (2009) delivered 40 trains of 3.9 s, separated by an intertrain interval of 26.1 s.

### *cTBS* and *iTBS*

Theta burst stimulation is a pattern of rTMS that induces synaptic plasticity by using bursts of high-frequency theta stimulation. In TBS, pulses are applied in three bursts at high frequency (50 Hz), with a burst interval of 200 ms (5 Hz, which is in the range of theta frequency). Theta burst stimulation requires less stimulation time and lower intensity to produce longer-lasting effects in the human cerebral cortex compared with other known rTMS protocols (Huang et al., 2005). There are two main patterns of TBS: continuous and intermittent. In cTBS, the pulses are delivered without interruption. In contrast, in iTBS, pulses are repeated every 10 s. In Chung (2015)’s study, it was noted that iTBS induces a facilitatory effect persisting for at least 15 min beyond the stimulation period, while cTBS decreases cortical excitability beyond the stimulation duration. Subsequent research, such as that conducted by McCalley et al. (2021), further explored this contrast. McCalley’s findings revealed that 1) 1200 pulses of iTBS significantly inhibit motor cortex excitability; 2) 3600 pulses of cTBS significantly increase motor cortex excitability; and 3) individual response to a given cTBS or iTBS protocol (e.g., 600 pulses) is inconsistent with the same individual’s response to another cTBS or iTBS protocol (e.g., 1200 pulses).

Two studies included in this review used cTBS and iTBS. Holczer et al. (2021) applied cTBS that contained 600 uninterrupted pulses given for 40 s (with a pattern of 3 pulses at 50 Hz in every 200 ms). The number of pulses was identical during iTBS, but the pattern consisted of 3 pulses in a train of 2 s given at 50 Hz, repeated every 10 s for 40 trains. However, Cheng et al. (2016) administered a 120-s train of uninterrupted bursts in cTBS and a 2-s train of bursts that was repeated every 10 s for a total of 570 s in iTBS.

### Online versus offline stimulation

All studies used either an online or offline design. Online effects are those that occur during stimulation and are typically disruptive in nature, whereas offline effects outlast the stimulation and are typically neuromodulatory in nature. In this review, we studied the effect of TMS treatment on cognition; hence, all the studies included utilized an offline design. Most of the tasks in the studies were executed at baseline and at the end of treatment. However, some of them collect behavioural data also in the middle of the treatment. For example, Levkovitz et al. (2009) conducted his task at baseline, visits 11 and 21, and Naim-Feil et al. (2016) evaluated the cognitive performance at baseline, after the first session of treatment (short-term), and immediately before the 20th TMS session (long-term). Others assessed cognitive performance at baseline, at the end of treatment, and some months later to perform a follow-up (Nadeau et al., 2014; Concerto et al., 2015).

### Intensity

The impact of TMS on a cluster of neurons depends on a variety of parameters, one of which is the intensity chosen. There are many options; the ones described in this review are: stimulating all subjects at the same intensity relative to the motor threshold (MT), and stimulating all subjects at the same absolute intensity.

#### Relative to the individual motor threshold

The stimulation intensity is set to a percentage of the individual MT to determine the corresponding intensity. Depending on the state of the muscle, there are two main MT: 1) the resting motor threshold (rMT), and 2) the active motor threshold (aMT). rTM is determined with the target muscle being completely relaxed and is defined as the amount of energy required to elicit movement in the contralateral hand (typically abductor pollicis brevis) 50% of the time. It refers to active motor threshold when the MT is determined during a slight tonic contraction of the target muscle with approximately 20% of the maximal strength. All the studies reviewed have determined the MT of the participant when the muscle was completely relaxed, except for Cheng et al. (2016), who used an active MT.

In this review, the majority of the studies used intensities between 100% and 120% relative to the individual MT. Kedzior et al. (2012), Kuroda et al. (2006), Nadeau et al. (2014), Şalçini et al. (2018), and Schaffer et al. (2020) used protocols in which the intensity was set at 100% of the individual MT. Levkovitz et al. (2009), Martis et al. (2003), Pallanti et al. (2012), Rostami et al. (2022), Vanderhasselt et al. (2009) and Yildiz et al. (2023) applied intensities of 110%, and Bahun et al. (2022), Concerto et al. (2015), Corlier et al. (2020), Kavanaugh et al. (2018), Levkovitz et al. (2009), Naim-Feil et al. (2016), and Rostami et al. (2022) (right) set at 120% relative to the individual MT. Spampinato et al. (2013) set an intensity of 130% of the rMT. However, Cheng et al. (2016) and Moser et al. (2002) set the intensity to less than 100%.

#### Fixed absolute intensity

Another option is to fix the same absolute intensity (a percentage of the maximum stimulator output) for all subjects. Normally, studies that use rTMS applied pulses at an intensity ranging from 60% to 65%. In cTBS studies, the pulses’ intensity is usually lower, around 40% of the absolute maximum stimulator output. Two studies, in this review, used this method. Bermpohl et al. (2006), utilizing LF-rTMS, set the stimulation intensity to a fixed level of 60% of a maximum stimulator output. However, in Holczer et al. (2021) study, cTBS and iTBS were used, so the stimulation intensity was set at 30% of the maximal stimulator output for all the participants.

### Localization method

Knowing the targeted area exact position is crucial for TMS further effect. There are various methods to find the right placement; the studies reviewed have used five different types: individual MRI and neuronavigation; 5-cm rule; Beam F3 method; anatomical landmark; and 10-20 system.

### Individual MRI and neuronavigation

MRI-guided neuronavigation was developed to achieve a precise localization method. Neuronavigation aligns the MRI scan acquired earlier in time with the head of the patient by measuring several points on the head with a tracker. The TMS coil and the head of the patient are also tracked. This allows neuronavigation software to accurately guide a TMS pulse to the intended neuroanatomical structure with millimeter precision.

In the present review, four studies used this localization method (Moser et al., 2002; Vanderhasselt et al., 2009; Cheng et al., 2016; Holczer et al., 2021). All of them defined the left DLPFC as a point between the junction of Brodmann area (BA) 9 and 46, involving the anterior of the middle frontal gyrus, by referral to each patient’s brain MRI scan. Afterward, precise coil positioning was supported by a TMS Neuronavigation. According to Moser et al. (2002), the target site was located at an average of 5.3 cm anterior and in a parasagittal plane from the point of maximum stimulation of the right abductor pollicis brevis muscle.

### Beam F3 method

**Fig. 2:**
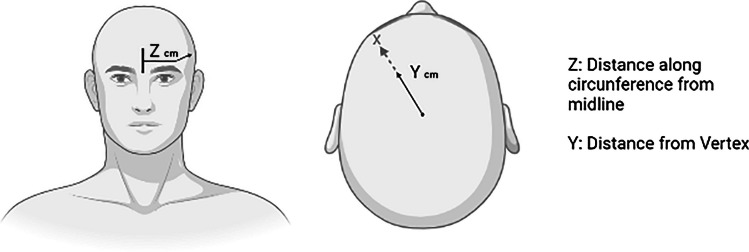
Two values outputted by the program to locate F3. Z is the distance to a point along the circumference from the centerline. Y is the distance from the vertex along a line intersecting point X

This method was developed to mimic neuronavigation without the cost associated with using those devices. In order to calculate the F3 position, the user has to enter three measurements into a computer program, 1) the distance from the tragus to the tragus, 2) the distance from nasion to inion and 3) the head circumference. The program outputs two values that are used to locate F3 location from the 10-20 system. These two measurements are the distance to a point (point x) along the circumference from the center line and the distance from the vertex along a line intersecting point x (Beam et al., 2009). Compared with the 10–20 system, this method is less time-consuming, because fewer measurements and calculations need to be done. One of the studies included in this review (Corlier et al., 2020) used this method to find DLPF (Fig. 2).

### 5-cm rule

This method was the most used among the studies included in this review (12 in total). It is so because of its ease to use in clinical settings. It consists of evoking a motor response in the first dorsal interosseous muscle of the contralateral hand, then moving 5 cm rostrally in the parasagittal plane (Herbsman et al., 2009).

Of the eleven studies that used this method, two of them slightly modified it. (Levkovitz et al., 2009) placed the coil 5.5 cm anterior to the motor spot. He argued that the coil was placed a little more anteriorly because the 5 cm was shown in many cases to result in placement over the premotor cortex rather than the relevant areas over the prefrontal cortex. In Naim-Feil et al. (2016) study, the coil was moved 6 cm anterior of the motor spot and placed over the PFC.

### Anatomical landmark

Kavanaugh et al. (2018) was the only one to use this method as a means to localize the structures stimulated. A two-coil device was used, and its centers (the point of highest magnetic field intensity) were positioned over the left DLPFC and the bilateral dorsomedial prefrontal cortex. This localization was possible thanks to manual-driven instructions that used the identification of anatomical landmarks frontal, for example, process of the zygomatic bone, temporalis muscle, and intersection of the midline and the intertragal line.

#### 10-20 EEG system

In Kedzior et al. (2012) study, the F3 position in the 10-20 system was used to locate the left DLPFC. This system is based on the relationship between the location of the electrode and the underlying area of the cerebral cortex. The numbers “10” and “20” refer to the fact that the distances between adjacent electrodes are either 10% or 20% of the total front-back or right-left distance of the skull. The 10-20 system has been increasingly applied for the positioning of TMS in cognitive neuroscience and psychiatric treatment studies since it is applicable at a low cost and does not require a previous neuroimaging process.

### Coil type

Several types of coils with different shapes and sizes have been developed intending to improve the stimulation. In this review, we can find mostly figure-of-eight coils, but there are also Hesed coil (H coil) and two coil devices.

### Figure-eight coil

The figure-of-eight coil provides a focused stimulation compared with the round coil. The electric field is at its maximum under its center (hot spot), where the two rings meet (Ueno & Sekino, 2021). Thereby, we can deliver localized and focal brain stimulation. Because of these advantages, 15 studies used this type of coil.

### Hesed coil (H coil)

The H coil enables effective stimulation of deep brain regions without inducing a much greater stimulation of superficial cortical regions (Zangen et al., 2005). When this coil is used, it is called deep TMS. Compared with the figure-eight coil, the new Hesed Coil (H- coil) produced an electric field that penetrates the cortex to 3 cm deep. There are several versions of the initial H coil; they are used depending on the treatment and the pathology.

In the present review, three studies used H coil. Bahun et al. (2022) utilized the H1 coil as well as Naim-Feil et al. (2016). In contrast, Levkovitz et al. (2009) used three different versions of H coil: the H1, which is designed to stimulate preferentially the left hemisphere; the H2 coil, which is designed to stimulate deep prefrontal brain regions bilaterally, with no lateral preference, and the H1-L coil, which is designed to stimulate unilaterally, exclusively in the left hemisphere.

### Two-coil device

The two-coil or double-cone coil is a new device composed of two large circular wings, fixated at a 120 angle. Among the diverse coils existent in the market, this one has demonstrated the best balance between the depth and focal length of the stimulus, because it is the most focal coil that can reach deep targets within the energy limit of standard TMS devices (Monteiro & Cantilino, 2019).

Kavanaugh et al. (2018) was the only one to use this device. In his study, two center-folded double coils were placed over the left DLPFC and bilateral dorsomedial prefrontal cortex, respectively.

### Control condition

In every TMS study, a control group should be used to guarantee that the general interference and auditory and tactile effects from the TMS stimulation are considered. There are several ways to deal with this difficulty. The ones shown in this review are 1) control condition based on sham or placebo TMS and 2) control condition based on stimulating alternative cortical sites. Another approach shown in this review is to recruit a group of healthy volunteers to evaluate the normal performance in the cognitive assessment but without receiving TMS stimulation.

### Control condition based on sham or placebo TMS

According to Loo et al. (2000), the ideal sham for TMS would have the following characteristics: 1) placement of the TMS coil identical to that used for treatment to obtain visual and tactile parity with the “real” treatment; 2) comparable scalp sensation arising from stimulation of superficial nerves and muscles; 3) similar acoustic artefact of TMS, time-locked to the scalp sensation; and 4) no physiological effect on the cortex. Achieving this type of control condition would be a reliable comparison. However, it is exceedingly difficult to deliver any magnetic stimulus from the same coil in contact with the scalp and powerful enough to produce scalp sensation and not depolarize cortical neurons.

Nine studies used this approach. Cheng et al. (2016) and Vanderhasselt et al. (2009) set the coil at 90 degrees to the skull; however, in Concerto et al. (2015), Spampinato et al. (2013) and Yildiz et al. (2023) studies, the sham stimulation was performed by angling the coil at 45 degrees to touch but not to stimulate the skull. Something similar occurred in Moser et al. (2002)’s study, where the figure-eight coil was positioned above the top of the skull with the handle placed against the head. Holczer et al. (2021) used a plastic block to elevate the coil from the scalp by 4 cm; therefore, the participants could still experience the same mechanical vibration as the real treatment and heard the clicking sound of the device without significant cortical stimulation. Kavanaugh et al. (2018) positioned the coil in the same place as in the treatment group but without delivery of any energy and simulated the sound of the discharging coils through speakers near the subject’s head. Nadeau et al. (2014) and Şalçini et al. (2018), all the same, used sham coil using the same procedure as the patient group. In the case of Nadeau et al. (2014), 2-cm-diameter EMG electrodes were positioned beneath the stimulating surface to deliver electrical stimulation to the scalp simultaneous to the sham treatment.

Although this control condition is a good option, it does not reliably mimic the peripheral sensations associated with the magnetic field change, which may interfere with the need to keep the subjects blind to the control condition (Bermpohl et al., 2006).

#### Control condition based on stimulating alternative cortical sites

Stimulating another area of the brain is another possible control condition. Yet, the area has to meet two conditions: 1) the distance from the target area should be at least 1.5–2 cm, and 2) this area cannot be involved in the cognitive network active during the task execution (Garcia-Sanz et al., 2022). Only one study included in this review used this method. Bermpohl et al. (2006) included mesial occipital rTMS as an active control condition that also controlled for nonspecific effects of rTMS, such as discomfort due to muscle contractions, head-tapping, and clicking sounds.

#### Control condition based on cognitive assessment with no TMS stimulation

In this control condition method, no TMS stimulation is delivered, and its effects (sound and muscle contractions) are not mimicked. In the case of Kedzior et al. (2012), Kuroda et al. (2006), Levkovitz et al. (2009), Naim-Feil et al. (2016), and Yildiz et al. (2023), healthy participants were recruited to evaluate their normal performance in the cognitive assessment tasks, but they did not receive any TMS treatment. The participants were screened and excluded if they had a potential current or historical major medical/psychiatric illness. All the healthy groups in the studies, except for the one in Kedzior et al. (2012), were matched by age and level of education with their corresponding treatment group. Kedzior et al. (2012), however, considered his healthy group as volunteers rather than control, because they were not matched with the patients. The healthy volunteers were considered participants in a parallel substudy, and their data were analysed separately.

### Cognitive task and effect of TMS treatment

#### Visuospatial function and recognition

The visuospatial function involves identifying stimuli and their location. This function activates different cortical areas, such as the V5 (Brodmamm area) superior parietal lobe, parieto-occipital junction, and premotor areas (Quental et al., 2009). Five studies evaluated this function in diverse ways. One of the tasks was the block design subtest, where the participants replicated a pattern that the examiner presented to them by using an increased number of identical blocks. This task was used by two studies: Nadeau et al., 2014; Bahun et al., 2022. Nadeau et al. (2014) combined this task with rTMS on the left and right DLPFC and did not find any significant difference between the baseline and 3 months later. However, Bahun et al. (2022), combining the block design subtest with dTMS on the left DLPFC found a significant main effect of time (*p* = 0.022). Rostami et al. (2022) and Levkovitz et al. (2009) used a different task: the Stocking of Cambridge. Participants had to copy the display of three coloured balls, making as few moves as possible to match the previous pattern. Rostami et al. (2022) used HF-rTMS and LF-rTMS on the left and right DLPFC, and a significant difference was achieved between pre-and post-treatment. Levkovitz et al. (2009) also found a significant difference between the baseline and the visit 21 (5 weeks) in the H1 and H1L-120% groups. Moser et al. (2002) stimulated the anterior portion of the left middle frontal gyrus with rTMS while executing the judgment of line orientation, consisting of matching angled lines to a set of 11 lines that were arranged in a semicircle and separated 18 degrees from each other. No significant difference was found. Finally, Kedzior et al. (2012) used fast frequency rTMS on the left DLPFC. Repeatable battery for the assessment of neuropsychological status (RBANS) was executed into two parts: 1) figure copy consisted of copying a geometric figure comprised of ten parts, and 2) line orientation was described previously. No significance was found in either of the tests.

### Executive function

It refers to the higher-level cognitive skills used to control and coordinate your other cognitive abilities and behaviours. Bahun et al. (2022), Kuroda et al. (2006), Moser et al. (2002), Nadeau et al. (2014), Şalçini et al. (2018), and Yildiz et al. (2023) used the Trail Making test (TMT), which is divided into two parts: the TMT-A and TMT-B. In each test, participants were asked to draw a line between 24 consecutive circles that were randomly arranged on a page. The TMT-A used numbers, whereas the TMT-B required the participant to switch between numbers and letters in consecutive order. Bahun et al. (2022) combining dTMS over the left DLPFC and TMT task detected a significant effect in the main effect of time TMT-time (*p* = 0.022). rTMS over the left DLPFC was used by Kuroda et al. (2006) and Şalçini et al. (2018), who found no significant difference. Yildiz et al. (2023) also applied rTMS over the left DLPFC and observed significant results before and after the stimulation (TMT-A *p* = 0.001; TMT-B *p* = 0.011). However, these results did not reach significance when the treatment group and sham were compared. In another study (Nadeau et al., 2014), this task was incorporated with left and right DLPFC rTMS stimulation, but no significance (*p* = 0.944) was found between the baseline and 3 months later. Moser et al. (2002) utilized rTMS on the anterior portion of the left middle frontal gyrus while executing the TMT task and WAIS R Digit Symbol. In the first one, a significant improvement (*p* < 0.05) in the follow-up of the TMT-B was found. In the second, which consisted of digit-symbolpairs followed by a list of digits, where the participants had to write down the corresponding symbol under each digit as fast as possible, no significance was observed. Concerto et al. (2015) used a different task: the Frontal Assessment Battery (FAB), which consisted of six tests that correspond to each specific cognitive or behavioural domain related to the frontal lobes. He used HF-rTMS over the left DLPFC, and no significant improvement was found between pre and post rTMS treatment. Spampinato et al. (2013) used the same test and protocol as Concerto et al. (2015), and no significance (*p* = 0.110) was found either.

### Phonemic fluency

Phonetic fluency involves strategic search and retrieval processes within orthographic or phonological networks and thus requires several higher-order functions. Pallanti et al. (2012) used LF-rTMS on the right DLPFC when participants executed the FAS verbal fluency test, which required an individual to orally produce as many words as possible, during 1 min, that begin with the letter F. The same procedure is repeated for the letters A and S. No significant difference was found in any of the groups (patients, *p* = 0.065; responders, *p* = 0.65; nonresponders, *p* = 0.43). Bahun et al. (2022) also executed the FAS verbal fluency test while stimulating the left DLPFC with dTMS. No significant effect (main effect of time, *p* = 0.085; main effect of modality, *p* = 0.988) was found. In another study (Martis et al., 2003), the Oral word association, in which participants were asked to say as many words as possible from a given category in a specified timeframe, was combined with left prefrontal stimulation using rTMS. Martis et al. (2003) found a significant improvement (*p* = 0.01) between baseline and post-rTMS. In two other studies, the Boston naming test, which consisted of identifying drawings of objects ranging from common to less familiar, was executed. Nadeau et al. (2014) and Moser et al. (2002) combined this task with the stimulation of the left and right DLPFC, and the anterior portion of the left middle frontal gyrus, respectively. None of them found a significant improvement. Moser et al. (2002) did another task, the sentence repetition test, in which the participant was required to repeat sentences of increasing difficulty and complexity directly after the examiner read them. No significant difference was found.

### Inhibition

Inhibitory control involves being able to control one's attention, behaviour, thoughts, and/or emotions to override a strong internal predisposition or external lure and instead do what is more appropriate or needed. Nine studies used the Stroop test, which required individuals to view a list of words that were printed in a different colour than the meaning word. Participants were asked to name the colour of the word, not the word itself, as fast as they can. Concerto et al. (2015) and Spampinato et al. (2013) combined this task with HF-rTMS over the left DLPFC. Concerto et al. (2015) found a significant effect (*p* = 0.0013) between T0 (baseline) and T1 (end of the rTMS), but no significance was found after 3 or 6 months. Spampinato et al. (2013) also found a great difference (*p* = 0.00004) between baseline and post-treatment. Three studies (Şalçini et al., 2018; Corlier et al., 2020; Yildiz et al., 2023) utilized rTMS to stimulate the left DLPFC. Corlier et al. (2020) identified a significant difference among responders and in the incongruent condition. Şalçini detected a significant difference (*p* < 0.05) between the baseline and the 20th session. Yildiz et al. (2023) observed noteworthy outcomes across all three categories when comparing before and after stimulation, along with significant results in neutral words when comparing the active and sham groups. In complex attention and cognitive flexibility, Schaffer et al. (2020) found an improvement (*p* < 0.01 and *p* < 0.001 respectively) using LF-TMS on the right DLPFC and supplementary motor area (SMA). There was no improvement in Martis et al. (2003), Moser et al. (2002), and Nadeau et al. (2014)’s studies, where the left prefrontal cortex, the anterior portion of the left middle frontal gyrus, and the left and right DLPFC were respectively stimulated with rTMS. Bermpohl et al. (2006) used another task called Affective go/no-go. This task consisted of several blocks presenting a series of words from two of three different affective categories: Positive, Negative, and Neutral. The participant was given a target category and was asked to select a word when it matches this category. He used LF-rTMS on the left and right DLPFC, and he only found a significant difference when comparing the right rTMS treatment and the control in depressed patients (*p* = 0.02) and when comparing the left rTMS treatment and control in improved patients (increase in error number *p* =0.05).

### Attention

This function was studied in this review with diverse tasks. Holczer et al. (2021) used the attention network task (ANT). The participant was shown cues in the form of one or two asterisks, which could be used to predict an upcoming target presentation and/or be informative of the target’s location. The cue was followed by the presentation of target arrows, which could appear individually or in a sequence of an array of five arrows. Participants were to respond by indicating directionally which way the central arrow was facing. Holczer et al. (2021) used cTBS and iTBS on the left and right DLPFC and no significant difference was found (*p* = 0.161). Another task was the vigilance task in which participants were asked to find and cross out either sixes or nines as fast as possible, which appeared randomly within 59 rows of single digits. It was used by Kavanaugh et al. (2018), combined with rTMS over the left DLPFC and over the bilateral dorsomedial prefrontal cortex and found no significant improvement. Levkovitz et al. (2009) and Naim-Feil et al. (2016) opted for using dTMS on the left DLPFC and assessed attention by utilizing rapid visual processing (RVP) and sustained attention to response task (SART), respectively. In RVP, digits from 2 to 9 appeared in a pseudo-random order, and participants were requested to detect target sequences of digits (e.g., 2-4-6, 3-5-7, 4-6-8). When the participant saw the target sequence, they had to push a button as quickly as possible. There was a significant difference between baseline and visit 21 in the groups H1, H2, and H1L-120%. In SART, participants had to withhold behavioural response to a single, infrequent target (often the digit 3) presented amongst a background of frequent nontargets (0-2, 4-9). A significant difference between baseline and single session (*p* = 0.036) and baseline and long-term treatment (*p* = 0.023) was achieved. Schaffer et al. (2020) chose to use LF-TMS over the right DLPFC and SMA in the continuous performance task, a test usually used where the subjects perform a constant-difficulty task for minutes without interruptions. Complex attention was found significantly different (*p* < 0.01) between the pre- and post-TMS treatment. Kedzior et al. (2012) used the RBANS when stimulating the left DLPFC with fast frequency rTMS. In this case, two subtests were executed: a) digit span and b) coding. No significance was achieved (*p* = 0.150) between the baseline and 20 days afterward. Squire in 1979 created a test now used by Martis et al. (2003) in his study, which combined with rTMS left prefrontal stimulation had no significant effect. Lastly, Nadeau et al. (2014) used a Paced auditory serial addition test in which single digits were presented every 3 s, and the participants had to add each new digit to the one immediately before it. rTMS was used over the left and right DLPFC. Comparing the results between the baseline and after 3 months, a significant difference was found (*p* < 0.0001).

### Set shifting/flexibility

Cognitive flexibility refers to the ability to switch between thinking about two different concepts. It also refers to the ability to adapt flexibly to our constantly changing environment. Cheng et al. (2016) and Yildiz et al. (2023) used the Wisconsin card sorting test (WCST) to measure this function. This test is to classify cards, which differ by three criteria: colour, shape, or number, with the experimenter changing the criterion at short intervals. iTBS and cTBS were used in Cheng et al. (2016)’s study over the left and right DLPFC. It was found that the patients receiving iTBS had a significant effect between baseline and post-treatment (total errors *p* = 0.027; total corrects *p* = 0.027). Yildiz et al. (2023) discovered significant results in the number of categories completed (*p* = 0.017), the total number of correct responses (*p* = 0.046), and the total number of false responses (*p* = 0.046) when comparing before and after the rTMS stimulation. Kedzior et al. (2012) used fast-frequency rTMS and chose to use the modified concept-shifting task (mCST), which consisted of 8 trials during which each participant had to cross out, as fast as possible, 10 numbers or 10 letters in ascending or descending order. The task incorporated concept shift and attention shift by changing the rules used to respond to the same set of stimuli. Both, the duration (*p* = 0.047) and the accuracy (*p* = 0.038) were significant between baseline and after 20 days of rTMS treatment. Another study (Vanderhasselt et al., 2009) created a switching task that contained three blocks. During the first block, participants, when they saw an illuminated push-bottom, had to remove their finger from the central push button and push out the light. In the second block, participants were instructed to press their foot on a pedal when they heard a buzzer. The third block, the double task condition, was an alternating switch block with 29 auditory and 28 visual stimuli that were randomly mixed. HF-rTMS over the left DLPFC was used, and a difference in the auditory and visual decision time (*p* < 0.05) between pre- and post-treatment was observed.

### Memory

Because there are a variety of subtypes of memory such as episodic, procedural, and working memory, studies have chosen different tasks to evaluate it. Kavanaugh et al. (2018) executed three tasks: 1) delayed word recall, where the participant had to verbally recall the words to the examiner; 2) word recognition, which consisted of a list of 30 words presented on a computer screen (15 from an original list) and the participant had to select YES or NO as to if the word was on the original list; 3) picture recognition, which consisted of 40 pictures presented on a computer screen (half from an original list) and the participant had to select YES or NO as to if the picture was on the original list. Kavanaugh et al. (2018) employed rTMS to stimulate the left DLPFC and bilateral prefrontal cortex while executing these tasks, resulting in a significant difference (*p* = 0.028) in the quality of episodic memory. Kuroda et al. (2006) opted for rTMS over the left DLPFC and used two tests to evaluate memory, the everyday memory checklist, a subjective measure of memory failure in everyday life, and the Wechsler memory scale revised (WMS-R), a neuropsychological test designed to measure different memory functions. Both tests were found significant between the pre- and post-rTMS treatment. The same task WMS- R was utilized by Martis et al. (2003) while applying rTMS over the left prefrontal cortex. A significant improvement (*p* < 0.01) in objective memory was observed. RBANS included four subtests: (a) List Recall involved, (b) List Recognition, (c) Story Recall, and (d) Figure Recall. These tasks combined with fast frequency rTMS over the left DLPFC were utilized in the Kedzior et al. (2012) study. Significant results were found (*p* = 0.030) in the immediate memory comparing the baseline and Day 20 of rTMS treatment. In another study, the Corsi block tapping test, in which participants were asked to repeat the sequence tapped by the evaluator on nine blocks, was executed. LF TMS over the right DLPFC combined with this task resulted in a significant difference (*p* = 0.011) between baseline and 3 weeks after. The California verbal learning test was used by Nadeau et al. (2014) when stimulating the left and right DLPFC with rTMS. No significant effect was found between baseline and 3 months of treatment. In Bahun et al. (2022) study, four different tasks were executed: the Auditory-Verbal Learning test (AVLT), the verbal-logical memory subtest from the Wechsler Memory Scale-Revised, and the Benton Visual retention test (BVRT). In AVLT participants were given a list of 15 words repeated over six trials and were asked to repeat them. During the Verbal-Logical Memory subtest, participants were given two forms of the short story that they had to recall. Finally, in the BVRT, participants were asked to reproduce each of the 10 designs from memory as exactly as possible on paper. These tasks combined with dTMS stimulation over the left DLPFC resulted in a general significant improvement. Moser et al. (2002) used the Rey auditory verbal learning test, which consisted of a list of words read aloud, and the participants had to recall as many of them as possible. Stimulation was executed on the anterior portion of the left middle frontal gyrus with rTMS. No significant difference was found between the baseline and follow-up. The paired associated learning (PAL) test consisted of memorizing in which box a particular pattern was located. Levkovitz et al. (2009) used this task while stimulating the left DLPFC with dTMS. A significant difference in H1 group was achieved. Participants in Schaffer et al. (2020) study completed the CNS Vital Signs (CNS-VS) computerized neurocognitive assessment, where tests of verbal and visual memory were executed. These tasks were recognition tests, however, not tests of recall. Stimulation over the right DLPFC and SMA with LF TMS did not result in a significant effect. Yildiz et al. (2023) used the verbal memory process test (VMPT) which involved the presentation of fifteen unrelated words verbally to patients for complete memorization. If patients fail to learn all the words, the examiner could repeat the test up to ten times. Thirty minutes after the learning session, patients were asked to recall as many words as possible. No significant result was found in this test.

### Working Memory

Working memory is a subtype of memory that is defined as the small amount of information that can be held in mind and used in the execution of cognitive tasks. The N- Back Paradigm is a task in which stimuli sequences are presented to participants. For each item in the sequence, individuals are required to determine if the presented stimulus matches the presented stimulus “n” items ago. Holczer et al. (2021) used cTBS and iTBS to stimulate left and right DLPFC in combination with the N back task. The 2back and 3back tasks were not significant (*p* = 0.249) in this study. Kavanaugh et al. (2018) opted for a Spatial Working memory (SWM) and a Numeric Working Memory using rTMS over the left DLPFC and bilateral dorsomedial prefrontal cortex. For the first one, a picture of a house with some windows lit was shown. Afterwards, a new picture of the house was presented with only 1 window lit, participants had to select if the single window was lit in the original picture. For the Numeric task, a series of 5 digits were presented one at a time. For the remaining 30 digits presented, the participant had to select YES or NO as to if the digit was in the original series. The quality of the working memory was found to be not significant (*p* = 0.249). Levkovitz et al. (2009) used dTMS on the left DLPFC while executing a SWM test. This task was a sensitive measure of dorsolateral and ventrolateral prefrontal cortex functioning. A significant improvement was found in groups H1, H2, and H1L-120%. In another study, stimulation over the left prefrontal cortex with rTMS was combined with WAIS III letter number span, which involves hearing a series of letters and digits, and reporting back the stimuli with the letters in alphabetical order, and digits in ascending numerical order, resulted in a significant improvement (*p* = 0.01). At last, Rostami et al. (2022) used HF and LF rTMS over the left and right DLPFC and executed SWM test, where the subjects had to “search through” several boxes presented on the screen, revealing what was inside and remembering the boxes that were already opened. A significant effect was found between pre-and post-treatment. Another task executed was a delayed matching of the sample. After a brief delay, the participant was shown a complex visual pattern, both abstract and nonverbal, followed by four similar patterns. The participant had to select a pattern which exactly matches the sample. Again, significant results were found between pre-and post-treatment. Bahun et al. (2022) and Yildiz et al. (2023) used the Digit span task; in this task, the ability to remember a sequence of numbers that appear on the screen was evaluated. Bahun et al. (2022) did not find any difference either forward (*p* = 0.471) or backward (*p* = 0.879), and neither did Yildiz et al. (2023) when comparing before and after rTMS or active to sham.

### Speed of information processing and motor functioning

Processing speed refers to the speed at which information can be sensed, perceived, understood, and responded to. Levkovitz et al. (2009) used dTMS over the left DLPC that was associated with the Reaction Time Task, in which participants had to respond with a specific response to the only stimulus that appeared, and no significant effect was found. Another study executed a simple and choice reaction time, in which there were multiple stimuli, and each stimulus requires a different response. An rTMS treatment was used over the left prefrontal cortex and no significant effect (*p* = 0.58) in attention and mental speed was observed. Finally, Schaffer et al. (2020) used LF TMS on the right DLPFC and SMA and chose the finger-tapping test, resulting in a significant difference (*p* < 0.05) between the pre-and post-treatment. In this task, the patient had to keep tapping an index finger on a table until the examiner instructed the patient to stop. A modification of this required the patient to perform a repetitive movement with the opposite hand, such as supination and pronation, while having them finger tap with the other hand.

## Discussion

This systematic review mainly investigates the role of different TMS protocols regarding cognitive control in depressed patients. Most of the selected studies supported the idea of cognitive improvement after a TMS treatment. Regarding the cognitive domains, inhibition, attention, set shifting/flexibility, and memory (and working memory) were the domains that showed the most improvement, having a significant positive effect in more than half of the tests. However, fewer evaluations of visuospatial function and recognition, executive function, phonemic fluency, and speed of information processing were found to be significant.

The enhancement of inhibitory control after TMS stimulation could be explained by several ideas. The first suggests direct strengthening of the inhibition network itself. The neurons in critical areas for this function, such as the pre-SMA and rIFG could fire more intensely. Communication pathways within this network also could be enhanced, allowing information to flow smoother and faster. Over time, repeated stimulation might even solidify connections within the network, making inhibition more automatic (Borgomaneri et al., 2020). Alternatively, the improvement might come from quieting down competing networks that hinder inhibition. There could be a reduction in background noise in brain regions that interfere with control, allowing the inhibition network to operate unhindered. There might even be unintended inhibitory networks accidentally suppressing control processes. TMS could fine-tune these networks, creating a more balanced and efficient system (Kim et al., 2012). Both scenarios align with observed changes in brain activity and chemistry after TMS but teasing out the exact mechanism remains a challenge. Unveiling this mystery requires further research, possibly using advanced neuroimaging to visualize brain activity and network interactions during and after stimulation.

TMS has emerged as a promising treatment modality for improving attention, primarily through its ability to modulate neural oscillations, particularly alpha rhythms (Lin et al., 2021). Alpha oscillations, crucial for attention regulation, are influenced by TMS application to specific brain areas, such as the DLPFC, where LF TMS increases alpha power, enhancing attentional focus. This effect is attributed to neural entrainment, wherein TMS synchronizes neural networks, leading to more efficient information processing and attentional control (Luber & Lisanby, 2014). The left dorsolateral prefrontal cortex serves as a key target for TMS due to its involvement in executive function, cognitive training, and working memory encoding, making it relevant for addressing attention deficits (Liu et al., 2020). Moreover, networks, such as the frontoparietal control network (FPCN) and default mode network (DMN), play critical roles in attention functions, with the FPCN coordinating goal-driven behaviour and the DMN facilitating introspective thought, indicating their involvement in attentional processes. In Bloch et al. (2010) study, 20-Hz rTMS was applied to the right DLPFC in 13 adults with ADHD. They used the Positive and Negative Affect Schedule (PANAS) questionnaire to assess emotions, including attention, hyperactivity, anxiety, and mood. The attention and hyperactivity scores were combined to create an overall ADHD score. Additionally, Visual Analog Scales (VASs) were employed to evaluate attention and mood states. The results indicated that right DLPFC rTMS significantly improved the overall ADHD score, with notable improvements in attention based on VAS scores. These findings collectively underscore the neural mechanisms underlying TMS-induced attention enhancement and its potential as a therapeutic intervention for attention-related disorders.

Studies examining HF-rTMS show promise in improving cognitive flexibility, particularly in individuals with MDD. Targeting the DLPFC with this technique has been shown to decrease perseverative and non-perseverative errors on the Wisconsin Card Sorting Test, a measure of cognitive flexibility (Boggio et al., 2005). These findings align with research suggesting the DLPFC plays a crucial role in this cognitive domain. rTMS is thought to modulate neural activity and connectivity in the stimulated area as well as influence various brain processes, such as metabolism and neurotransmitter activity (Breukelaar et al., 2017; Li et al., 2017; Menon & D’Esposito, 2022). These changes might ultimately lead to improved brain function, allowing individuals to switch between mental sets and adapt to new information more effectively, thereby enhancing cognitive flexibility. However, further research is needed to fully understand the mechanisms at play and optimize rTMS protocols for this purpose.

While the exact mechanisms are still being unraveled, research suggests that TMS influences memory through its ability to modulate neural networks. One way that it might work is by enhancing communication and activity in brain regions crucial for memory formation. In the Plas et al. (2021) experiment, 1Hz rTMS stimulation was delivered to the left DLPFC during episodic memory encoding. Simultaneously recorded EEG data indicated that the stimulation strengthened event-related power decreases in the beta frequency band in posterior areas. This was represented by higher beta power before word onset and lower beta power after word onset in the DLPFC group compared with the sham group. Taken together, the results showed a decrease in beta power that could indicate increased activity in areas crucial for memory encoding.

DLPFC also is accepted to exert executive control on the Working Memory (WM) system, although its exact functional specialization remains controversial. It has been associated with the encoding and retrieval phases of WM tasks, with consistent evidence implicating parietal areas in the actual storage of information (Balconi, 2013). The DLPFC may regulate the signal-to-noise ratio of the parietal cortex to enable increased storage capacity. HF-rTMS can entrain endogenous alpha frequency in the stimulated area, suppressing distracters and thus enhancing WM capacity. Other evidence points to the executive role of DLPFC in suppressing interfering task-irrelevant information and updating goal representations based on context information or task-related demands (Edin et al., 2009). In a study conducted by Bagherzadeh et al. (2016), an offline rTMS stimulation was used to show improvements in WM after multiple TMS treatment sessions in healthy individuals. The findings showed that stimulating the left DLPFC led to improved performance in tasks that required updating and monitoring of spatial information in the S2B task, which confirmed the central executive role of the DLPFC in WM. There also was an increase in capacity observed in the DSP task, which suggests that the left DLPFC could also play a part in controlling pure storage by filtering out distractions and exerting top-down control. This elucidates the potential role of TMS stimulation in the DLPFC to enhance working memory performance.

The observed not significant results in visuospatial functions and phonemic fluency also could be explained by the brain network of these functions. To understand the neural correlate in the brain of the visuospatial functions, a lot of research has been done using different neuroimaging (Cohen et al., 1996; Mellet et al., 1996; de Graaf et al., 2010) and Non-Invasive brain Stimulation (NIBS) methods (Valero-Cabré et al., 2008). These studies indicated that only the right, not the left, fronto-parietal network is relevant for this function. This hemispheric dominance was corroborated by a 2007 study in which TMS and fMRI were executed simultaneously during a visuospatial judgment task (Sack et al., 1991).

Concerning phonemic fluency, studies indicate that this function depends on the left frontal structures, particularly it depends more strongly on the left perisylvian regions (Biesbroek et al., 2021). Moreover, anodal tDCS stimulation over the Broca’s area, a region implicated in speech production and located in the inferior frontal gyrus, has shown improvement in verbal fluency in general (Cattaneo et al., 2011), suggesting that this region also may be implicated in phonemic fluency. Most of the protocols used in this review, while executing a visuospatial function and phonemic fluency tests, focused on the left DLFC, not on the specific regions of these functions. This could be a possible explanation for why these functions did not improve after TMS treatment.

The executive function in this review was mostly assessed by the Trail Making Test and the Frontal assessment battery and no significant effect was found after TMS treatment. Concerning the TMT, this result contradicts the claims of Martin et al. (2017), in which a quantitative analysis of 18 sham-controlled studies was executed resulting in an improvement in performance on the Trail Making Test parts A and B. Nevertheless, we cannot disentangle whether the different findings between Martin et al. study and our review occurred due to differences in TMS protocols, analysis (systematic review vs. meta-analysis), or study selection.

Regarding the speed of information processing and motor functioning, only three papers assessed this function utilizing subtests belonging to a battery of cognitive tests. This function was not given a significant role in the studies included in this review and, therefore, is not open to meaningful interpretation. A larger sample size and a consistent test may help to clarify the result found in this study.

It has been demonstrated that MDD patients who receive transcranial magnetic stimulation report positive improvements in their cognitive abilities and depressive symptoms (Gaynes et al., 2014). In agreement with the reviewed articles, there does not seem to be a direct link between this cognitive enhancement and the improvement in mood, indicating that rTMS may independently affect depressive symptoms and cognitive function.

Previous research in cognitive neuroscience has emphasized how particular brain networks and their connectivity play a critical role in shaping cognitive outcomes, such as memory (Serafini et al., 2015; Anderson et al., 2016). Notably, there is a significant link between the hippocampus and the frontoparietal network (FPN), with studies emphasizing the importance of their interaction in retrieval episodic memory (Bridge et al., 2017). This relationship sheds light on the potential neural pathways affected by rTMS. The primary target of rTMS, the DLPFC, is a key component of the FPN. Magnetic stimulation to the DLPFC may enhance connectivity within the FPN, potentially increasing the interconnectivity strength between the FPN and the hippocampus, thereby improving memory. Additionally, the precuneus, another major component of the FPN, plays a significant role in cognitive function, modulating both the FPN and the default mode network (DMN). rTMS treatment to the DLPFC may optimize precuneus activity within the FPN, resulting in heightened connectivity strength between the precuneus and the DMN. This enhanced connectivity could contribute to increased attentiveness and processing speed (Kim et al., 2019). Therefore, cognitive functions appear to be more associated with an increase in the excitability of the DLPFC and an enhancement of functional connectivity with the hippocampus and precuneus.

Concerning the neurobiological effects on depressive symptoms, rTMS appears to influence the metabolic activity of the anterior cingulate cortex (ACC), a structure implicated in the modulation of emotional behaviour and involved in evaluating the emotional significance of events (Drevets et al., 2008). During challenging emotional experiences, depressed patients exhibit abnormal activity in the rostral anterior cingulate cortex (rACC). This dysfunction is accompanied by difficulties in recruiting regions crucial for cognitive control and emotional regulation, such as the DLPFC and dorsal anterior cingulate cortex (dACC). Additionally, there are heightened amygdalar responses when dealing with tasks related to negative information (Pizzagalli, 2011; Pizzagalli & Roberts, 2022). The salience network (SN), comprising key nodes such as the dACC and the frontoinsular cortex, as well as subcortical regions, such as the amygdala and ventral tegmental area, plays a crucial role in detecting personally relevant and rewarding stimuli. It integrates external and internal cues of emotional, homeostatic, and cognitive nature, guiding appropriate behavioral responses. Research indicates reduced connectivity within the SN in individuals with MDD, correlating with symptom severity. Moreover, studies show heightened activation in SN nodes, such as the dACC, insula, and amygdala, in depressed individuals exposed to negative stimuli, suggesting dysfunction in salience processing, consistent with depressive tendencies towards negative self-perception and interpretations of external events (Anderson et al., 2016). Effective antidepressant treatment is associated with increased activation in the left DLPFC, a region often found to be hypoactive in MDD. Conversely, the subgenual cingulate, observed to be hyperactive in MDD, tends to show decreased activity with antidepressant response. Notably, rTMS-induced decreases in subgenual activity suggest a potential mechanism for the antidepressant effects of DLPFC-targeted rTMS, indicating a remote suppression of activity in limbic regions (Fox et al., 2012). Thus, the emotional effect is believed to be linked to the amygdala, SN network, and cingulate cortex.

Overall, most of the mentioned studies were conducted over the DLPFC. The rationale of this target is related to the abnormal activations of these regions in MDD and the crucial role that these regions play in cognitive functions, as previously described. However, one of the reviewed studies targeted the Supplementary Motor Area, besides DLPFC. This region was chosen because it has received attention in the treatment of anxiety-related symptoms and OCD (Mantovani et al., 2010), and in treating cognitive impairments and depressive symptoms among individual vascular dementia, as well, as specifically improving visual-spatial processing (Cona et al., 2017). It cannot be concluded that this area could be targeted alone for the treatment of cognitive deficits in MD as it was stimulated at the same time as the DLPFC.

Notably, not all participants in the studies were medication-free during its completion. Various groups were actively using antidepressants, and some also were prescribed hypnotics. This raises the question of whether these medications played a role in the observed cognitive enhancement among patients. Notably, existing literature suggests that medications can have both positive and negative effects on cognitive control and psychomotor functions, potentially interacting with TMS (Boggio et al., 2005; Hunter et al., 2019). For instance, Minzenberg & Leuchter (2019) conducted an empirical literature review to test how psychotropic drugs affect cortical excitability and plasticity by using diverse TMS paradigms. The findings indicated that antidepressants with potent 5HT transporter inhibition reduce excitability and alter plasticity, while those with other mechanisms of action generally lack these effects. Although, in the reviewed studies, some groups were medication-free, whereas others continued with their prescribed medications; all of them showed improvements in certain cognitive functions. It is noteworthy that the inclusion criteria for these studies mandated the maintenance of psychopharmacological treatment unchanged for at least 2 weeks preceding the application of TMS. This criterion was established to isolate the effects of TMS from the potential influence of recent changes in medication. Even so, further research is needed to address the question regarding the interaction of antidepressant drugs with TMS treatment in cognitive functions.

TMS has shown promise in treating cognitive deficiencies associated with major depressive disorder, but further research is needed to determine the best course of action. A more nuanced approach is required to confirm the effectiveness of TMS and prepare the way for its ideal application in clinical practice due to the heterogeneity of current research, which has inconsistent protocols and unclear results.

Addressing the constraints of the current research framework is a crucial next step. It is possible to conduct a strong meta-analysis that statistically synthesizes data from previous research to offer a more reliable assessment of the total effect of TMS on depression-related cognitive performance. Furthermore, performing subgroup analyses within the study can assist in identifying particular elements, such as differences in TMS protocols, patient characteristics, and used cognitive evaluations, that may be accountable for the noted variability in results. This would help to improve our comprehension of the various reactions to TMS and direct the creation of more focused and uniform research methods in the future.

It becomes essential to create the “ideal” clinical trial to strengthen the amount of evidence supporting TMS as a treatment for cognitive deficiency. This trial should incorporate several key components. First, utilizing double-blind sham stimulation as a control group ensures that the study is blinded, meaning neither researchers nor participants know who receives the real treatment or the placebo. This safeguards against biases and allows researchers to isolate the true effects of TMS beyond potential influences of expectation or placebo effects. Second, employing a diverse battery of cognitive tasks sensitive to various aspects of cognitive control (such as attention, working memory, and decision-making) offers a more comprehensive picture of how TMS impacts different cognitive domains. Finally, including a long-term follow-up period after the intervention allows researchers to assess whether observed improvements in cognitive function are sustainable over time. By implementing these essential elements, future trials can provide more definitive evidence for the efficacy of TMS and clear the way for its responsible integration into clinical practice.

## Conclusions

The results of the current systematic review provide evidence supporting the cognitive enhancing effect of TMS treatment when administered in depressed patients. Specifically, cognitive effects were observed in improving performance in inhibition, attention, set shifting/flexibility, and memory (and working memory). This significant enhancement of cognitive control may then be considered a treatment for cognitive deficits in patients suffering from MDD. Notwithstanding, these promising results support the need for future research in this field to clarify the actual TMS protocol divergence and the specificities of stimulation localization.

### Limitations

Studies included in this systematic review need to be considered with respect to the following limitations. First, the investigations incorporated different tests for the evaluation of a cognitive domain; therefore, the measurements and outcomes differ from each other. They assessed patients at different timelines: some cognitive tests were executed just one day after TMS treatment while others were executed 3 weeks after the treatment. Also, some of the studies did not include large sample size, giving insufficient power to detect significant changes. Second, the application of different stimulation protocols (i.e., intensity, frequency, pulses per session), the use of different TMS coils and the possible difference in the targeting of the stimulation need to be considered when evaluating the overall efficacy of TMS in cognition. Third, it has to be considered the variability of age in each treatment group. It has been shown that older adults, while having similar antidepressant responses to younger adults, respond more slowly (Cotovio et al., 2022). Thus, the TMS treatment duration of the studies could have been not enough for advanced ages. Lastly, most of the specified studies lacked a control group, which compromises validity and excludes the possibility of conclusions about causality. Numerous studies included in this review did not have a matched clinical group or matched healthy control group; instead, healthy volunteers were used as a control group who did not receive stimulation but performed only the cognitive tasks.

We also acknowledge some limitations in this systematic review, including selection bias, since our search algorithm only included publications specifically reporting TMS effect on cognitive control, and the database used, Google Scholar, has an extensive bibliography that is difficult to screen. We also acknowledge language bias , since we only included articles in English; however, they cover most of the current literature. Besides, our review results are only generalizable to analyzing depressed adults’ control cognition. Regarding this review, it was quite difficult to select the appropriate studies because of the restricted literature on this topic.

## Data Availability

All data supporting the findings of this systematic review are available within the paper. The data that support the findings of this study are derived from previously published studies, which have been cited and listed in the reference section of this paper.
